# Editorial: The role of immune cells in hepatic ischemia reperfusion

**DOI:** 10.3389/fimmu.2022.1075984

**Published:** 2022-11-04

**Authors:** Bibo Ke, Helong Dai, Qiang Wei, Zhiyong Guo, Tao Qiu

**Affiliations:** ^1^ The Dumont-UCLA Transplant Center, Division of Liver and Pancreas Transplantation, Department of Surgery, David Geffen School of Medicine at the University of California at Los Angeles, Los Angeles, CA, United States; ^2^ Department of Kidney Transplantation, The Second Xiangya Hospital of Central South University, Changsha, China; ^3^ Department of Hepatobiliary and Pancreatic Surgery, The Center for Integrated Oncology and Precision Medicine, Affiliated Hangzhou First People’s Hospital, Zhejiang University School of Medicine, Hangzhou, China; ^4^ Organ Transplant Center, the First Affiliated Hospital, Sun Yat-sen University, Guangzhou, China; ^5^ Department of Organ Transplantation, Renmin Hospital of Wuhan University, Wuhan, China

**Keywords:** liver, ischemia reperfusion injury, immune cells, macrophages, innate lymphoid cells, natural killer cells, hepatic stellate cells

We are honored to accept twelve articles on the Research Topic of “*The role of immune cells in hepatic ischemia-reperfusion*,” which have received widespread interest. Hepatic ischemia-reperfusion (I/R) injury is a pathological process involved in oxidative stress-induced cellular damage and immune activation during liver resection and transplantation. We have known that the generation of reactive oxygen species (ROS) from oxidative stress may be critical mediators during hepatic IR. However, there is growing interest in the roles of immune cells in IR-triggered liver inflammation and injury.

Liver resection and transplantation are the most effective treatments for patients with hepatocellular carcinoma. However, there is still a high recurrence rate after surgery, of which liver IR injury is closely related to liver cancer recurrence. Immune activation is crucial in the inflammatory damage process of liver I/R. The intense and sustained inflammatory response promotes tumor recurrence by activating tumor cell proliferation, adhesion, invasion, angiogenesis, and immune escape by many key inflammatory components. Targeting critical immune and inflammatory signaling pathways reduces I/R-induced liver inflammation and injury and prevents tumor recurrence after liver surgery, suggesting a promising win-win strategy (Chen et al.). The severity of I/R injury in the liver is primarily determined by the ratio of M1 to M2 macrophages. Hepatic I/R injury triggers the release of Damage-associated molecular patterns (DAMPs) that strongly activate the NLRP3 inflammasome. This essential molecule functions as a critical component of the innate immune response in hepatic macrophages. Increasing autophagy in hepatic macrophages can effectively alleviate liver I/R injury with depressed NLRP3 inflammasome activation (Wu et al.).

Moreover, macrophages are partially differentiated into M2 macrophages toward an anti-inflammatory phenotype and maintain environmental homeostasis. A novel study by Zhang et al. found that group 2 innate lymphoid cells (ILC2s) could induce polarization of M2-type CD45^+^CD11B^+^F4/80^high^ macrophages and that ILC2s proliferation was regulated by stimulation of exogenous IL-33 to exert a protective effect during hepatic I/R. This regulatory mechanism was found in the liver and the spleen. M1 macrophages are pro-inflammatory and play an essential role in activating liver inflammation during I/R. Liraglutide, a glucagon-like peptide-1 analogue, significantly inhibited the polarization of M1-type macrophages during hepatic I/R injury *via* the GLP-1 receptor, thereby improving hepatic I/R injury (Li et al.). The metabolic roles of intestinal microorganisms can produce substances with anti-hepatic I/R injury. Intestinal microorganisms break down inulin into short-chain fatty acids, one of the metabolites of which, propionic acid (PA), has a pro-inflammatory effect on macrophages. Inulin helps to regulate the gut microbes to maximize their effect. PA enters the portal vein to ameliorate I/R injury in the liver effectively. More importantly, PA directly inhibits TLR-4-HMGB1-mediated inflammatory responses in macrophages (Kawasoe et al.).

During hepatic I/R, DAMPs eventually lead to neutrophil activation and infiltration into the liver. The formation of neutrophil extracellular traps (NETs) released by neutrophils is critical in triggering an inflammatory response by releasing related enzymes and activating the complement system. NETs can also activate platelets leading to systemic immune thrombosis and organ damage. Neutrophils and NETs interact with other immune components of the tumor microenvironment in the transplanted liver to promote tumor progression (Kaltenmeier et al.).

Natural killer (NK) cells play a pivotal role in activating liver immune cells after reperfusion. NK cells can be recruited to the liver, increasing pro-inflammatory cytokine secretion and inducing early infiltration of neutrophils to exacerbate an inflammatory injury. Donor-derived NK cells are also gradually replaced by recipient NK cells after allogeneic liver transplantation, eventually exerting the negative effect of immunological rejection. NK cell depletion, inhibition of NK cell activation receptors, or blockade of signaling pathways for NK cell maturation can effectively reduce liver I/R injury (Huang et al.).

Hepatic stellate cells (HSCs) regulate liver I/R injury during the injury and repair/regeneration phases, depending on regulating different pathways and molecules. HSCs are activated and respond to signals from Kupffer cells during hepatic I/R. HSCs promote early I/R-induced injury by activating the ROCK-mediated hepatic microenvironment, ET-1 signaling, and the TNF-α-triggered inflammatory cascade. HSC-derived MMPs exacerbate injury by destroying ECM and recruiting leukocytes. However, the role of HSCs in the repair and regeneration phase deserves attention. The induction of HSC activation and proliferation by hepatic Kupffer cells also has a vital role in this process (Peng et al.). HSCs are the primary source of repairing myofibroblasts after injury. Shi et al. first reveal that phosphorylation of mixed lineage kinase domain-like protein (p-MLKL) is expressed in the periportal area. Activation of p-MLKL induces necroptotic cell death after liver transplant reperfusion. They also demonstrate that p-MLKL activates fibroblasts, a primary cell type that can effectively predict early graft injury during hepatic I/R.

The therapeutic effect of bio-nanomaterials on I/R injury of the liver has shown advantages over conventional molecules in various aspects and has good efficacy. Huang et al. showed that the Prussian blue (PB) is a good scavenger of ROS, which can reduce ROS production in hepatocytes and macrophages caused by various stimuli. PB reduces neutrophil infiltration, promotes M2 macrophage polarization, and ameliorates Hepatic I/R injury. Moreover, PB has good biocompatibility compared to other nanomaterials, suggesting that PB may be a potential therapeutic agent in managing hepatic I/R injury. Liggett et al. showed that steatotic donor livers are more susceptible to I/R-induced liver damage during transplantation. Type 1 Natural Killer T-cells (NKT1 cells) responses to endogenous lipid antigens mediate the exacerbation of I/R injury in the liver. Oral administration of N-acetylcysteine significantly reduced liver steatosis and downregulated CD1d to block NKT cell activation and reduce IFN-γ levels by using a reliable high-fat diet mouse model of liver I/R injury, thereby reducing damage. This study provides novel insights into the interplay between liver metabolism, I/R injury, and immune cells. In another study of steatotic donor livers, mice on a high-fat diet were found to significantly increase in type 1 Innate lymphoid cells (ILC1s) populations but not conventional natural killer cells after I/R injury, and ILC1 promotes inflammatory injury through T-bet-dependent forms of IFN-γ and TNF-α secretion. Furthermore, ILC1s is an intrinsic inflammatory effector subgroup in fatty liver. Targeting this immune subgroup provides evidence of the future use of marginal donor liver transplantation in the steatotic liver (Kang et al.).

The role of immune cells in hepatic I/R injury has been increasingly appreciated, particularly affecting innate immunity and adaptive immunity during hepatic I/R injury. However, the complex regulatory mechanisms between immune cells and hepatocytes in IR-triggered liver inflammation and injury are still unclear. Under this research theme, we have gained an initial understanding of the function of immune cells. Further study needs to identify the molecular regulators of immune activation and provide novel therapeutic approaches targeting immune cells for treating I/R-induced liver inflammatory injury.

## Author contributions

TQ: writing the original manuscript. BK: writing and revising the manuscript. HD, QW, and ZG: supervision. All authors contributed to the article and approved the submitted version.

## Conflict of interest

The authors declare that the research was conducted in the absence of any commercial or financial relationships that could be construed as a potential conflict of interest.

## Publisher’s note

All claims expressed in this article are solely those of the authors and do not necessarily represent those of their affiliated organizations, or those of the publisher, the editors and the reviewers. Any product that may be evaluated in this article, or claim that may be made by its manufacturer, is not guaranteed or endorsed by the publisher.

